# Sorafenib and edaravone protect against renal fibrosis induced by unilateral ureteral obstruction via inhibition of oxidative stress, inflammation, and RIPK-3/MLKL pathway

**DOI:** 10.1007/s00210-024-03146-z

**Published:** 2024-06-14

**Authors:** Mohamed A. Abou Taha, Fares E.M. Ali, Ibrahim G. Saleh, El-Sayed Akool

**Affiliations:** 1 Department of Pharmacology and Toxicology, Faculty of Pharmacy, Al-Azhar University Assiut Branch, Assiut, 71524 Egypt; 2 Department of Pharmacology and Toxicology, Faculty of Pharmacy, Al-Azhar University, Cairo, Egypt; 3 Department of Pharmacy Practice, Faculty of Pharmacy, Sinai University, Kantara, Ismailia Egypt

**Keywords:** Unilateral ureteral obstruction, Edaravone, Sorafenib, Renal fibrosis

## Abstract

Renal fibrosis is the common endpoint of nearly all chronic and progressive nephropathies. Cell death and sterile inflammation are the main characteristics of renal fibrosis, which can lead to end-stage renal failure. The inflammatory reaction triggered by tissue damage is strongly related to necroptosis, a type of caspase-independent, regulated cell death. Using an animal model of unilateral ureteral obstruction (UUO), the anti-fibrotic effects of sorafenib (SOF), a multi-kinase inhibitor, and edaravone (EDV), a potent antioxidant and free radical scavenger, were examined in rats with obstructive nephropathy. Experimentally, animals were divided randomly into five groups: sham; UUO; UUO + SOF (5 mg/kg/day, P.O.); UUO + EDV (20 mg/kg/day, P.O.); and UUO + SOF + EDV groups. The kidney function biomarkers, oxidant/antioxidant status, renal mRNA expressions of TNF-α, collagen-1α, protein expressions of RIPK-1, RIPK-3, MLKL, caspase-8, HYP, MPO, and TNF-α were all significantly modulated by UUO. Administration of either SOF or EDV significantly attenuated cellular and molecular changes induced by UUO. Also, histopathological changes were improved. Moreover, SOF in combination with EDV, significantly improved UUO-induced renal fibrosis compared with each drug alone. Collectively, administration of either SOF or EDV or both of them significantly attenuated the rats with obstructive nephropathy, possibly by blocking the RIPK-3/MLKL necroptotic pathway and suppressing renal oxidative stress and inflammation.

**Supplementary Information:**

The online version contains supplementary material available at 10.1007/s00210-024-03146-z.

## Introduction

Chronic kidney disease (CKD) is a public health concern that results in high morbidity and a growing financial burden. Every year, CKD claims the lives of one million people and affects 10 to 15% of the global population (Kovesdy 2022). Renal fibrosis is the common endpoint of almost all chronic and progressive nephropathies (Moeller et al. 2021). Fibrotic illnesses are widespread, come in various shapes, and can be fatal. Over time, the renal function of all individuals with chronic renal disease gradually deteriorates. The process is typically irreversible, resulting in end-stage renal failure, necessitating kidney transplantation or lifelong dialysis (Panizo et al. 2021). The last pathway that connects all kidney disorders to chronic renal failure is progressive tubulointerstitial fibrosis. The most prominent characteristic of tubulointerstitial fibrosis is excessive extracellular matrix (ECM) deposition, notably the presence of collagenous fibers. Inflammatory cells such as mast cells, dendritic cells, lymphocytes, and macrophages commonly infiltrate the tubulointerstitial tissues and cause tubulointerstitial fibrosis (Liu 2011; Bülow and Boor 2019). Unilateral ureteral obstruction (UUO) has become a crucial model for investigating the processes behind renal fibrosis and assessing the effectiveness of various treatment strategies to treat renal illness. Most of the previous studies refer to the rodent model of UUO as representative of human renal disease mechanisms (Martínez-Klimova et al. 2019; Prieto-Carrasco et al. 2021).

Necroptosis is a brand-new kind of controlled cell death that is caspase-independent. Necroptosis is mediated by the development of the receptor-interacting serine/threonine-protein kinase 1 (RIPK1)/receptor-interacting serine/threonine-protein kinase 3(RIPK3) necrosome and includes the receptor-interacting protein kinases RIPK1, RIPK3, and mixed lineage kinase domain-like protein (MLKL). When RIPK1 phosphorylates RIPK3, phosphorylated RIPK3 activates MLKL, which ultimately causes a breach in the plasma membrane and subsequent releases of proinflammatory cellular components. In accordance with previous studies, RIPK3/MLKL-mediated necroptosis causes interstitial fibrosis in connection with tubulointerstitial damage (Dai et al. 2020; Popper et al. 2019).

Edaravone (EDV) is a strong antioxidant that scavenges free oxygen radicals (Takei et al. 2017). Edaravone has recently been licensed to treat adult patients with amyotrophic lateral sclerosis. Additionally, it has been used to treat motor neuron diseases with progressive neurodegenerative disorders accompanied by cerebral stroke and muscular atrophy (Ali and Baker 2017; Dash et al. 2018; Bailly 2019). It has also been suggested to treat various other illnesses, including sepsis, testicular injuries, bladder injuries, liver injuries, and retinal injuries (Kikuchi et al. 2012; Bailly 2019). Many experimental and clinical studies have also revealed that EDV might protect against kidney injury (Fu et al. 2020; İnce et al. 2019; Zhang et al. 2017).

Multi-kinase inhibitor sorafenib (SOF) was originally authorized to treat people with liver and kidney cancer (Llovet et al. 2008). In addition, SOF may be useful in the management of lung and liver fibrosis, according to earlier studies (Mejias et al. 2009; Reiberger et al. 2009; Chen et al. 2011; Zhang et al. 2013). Also, SOF has been shown to reduce tissue damage and inflammation in two disease models caused by RIPK-dependent cell death, indicating that SOF may have therapeutic potential by targeting necroptosis (Martens et al. 2017).

It is critical to develop preventive measures since chronic kidney injury results in irreversible lesions, such as renal interstitial fibrosis. So, this study was designed to describe and explore the therapeutic benefits and potential underlying the protective mechanisms of SOF and/or EDV against chronic obstructive nephropathy experimentally.

## Materials and methods

### Animals

Adult male Wistar Albino rats (10 weeks, 200 ± 10 g) were obtained from the animal house at the Faculty of Pharmacy, Al-Azhar University, Assiut, Egypt. Animals were housed in controlled conditions (23 ± 2 °C, 60 ± 10% humidity, 12/12 h dark/light cycle). The animals were housed (five rats per plastic cage). They were given access to a standard diet (19% proteins, 6% fibers, 3.5% lipids, and 6.5% ash) and tap water. They were harbored for 1 week for acclimatization before the start of experiments. The research protocol was approved by the research ethics committee of the Faculty of Pharmacy, Al-Azhar University, Assiut (Approval No. ZA-AS/PH/23/C/2023), in accordance with the guidelines of the principles of Laboratory Animal Care (NIH Publications No. 85-23, updated 2011). This study aligns with the ARRIVE Guidelines for reporting in vivo experiments (Percie du Sert et al. 2020).

### Drugs, kits, and chemicals

SOF (Nexavar, Bayer, Germany) and EDV were obtained from Sigma-Aldrich (St. Louis, MO, USA). Colorimetric kits, creatinine (Cat. No. 234,000), urea (Cat. No. 318,001), uric acid (Cat. No. 323,000), magnesium (Cat. No. 285,001-P), and total protein (Cat. No. 310 001), were from Spectrum company, Germany. ELISA kits, interleukin-1β (IL-1β) (Cat. No. CSB-E08055r), hydroxyproline (HYP) (Cat. No. CSB-E08838r), and tumor necrosis factor-alpha (TNF-α) (Cat. No. CSB-E11987r; RRID: AB-2,936,876), were from Cusabio, China. RIPK-1 (Cat. No. YPA1018), MLKL (Cat. No. YPA2507), anti-β-actin antibody (Cat. No. BTL1027), anti-heme-oxygenase-1 (HO-1) (Cat. No. YPA1919), and anti-nuclear factor erythroid 2-related factor 2 (Nrf-2) (Cat. No. YPA1865) were from Biospes, China. RIPK-3 (Cat. No. E-AB-60,962) and anti-cysteine-aspartic acid protease 8 antibody (caspase-8) (Cat. No. E-AB-62,025) were from Elabscience, USA. Alkaline phosphatase-conjugated secondary antibody (Santa Cruz, USA), anti-myeloperoxidase (MPO) (Invitrogen, USA, Cat. No. PA5-16672; RRID: AB-11,006,367), BCIP/NBT detection kit (Genemed Biotechnologies, USA), and goat anti-rabbit secondary antibody were also used. RNA extraction kit and c-DNA synthesis kit were obtained from Vivantis Technologies, Malaysia. All other chemicals were bought from regionally trusted suppliers and were of the analytical grade.

### Animal model of renal fibrosis

In a sterile and temperature-controlled environment, all surgical procedures were performed. Induction of renal fibrosis was conducted by UUO for 21 days (Shimizu et al. 1998). On the fourth day of the experiment at 8:00 am, rats underwent ketamine/xylazine anesthesia (100 mg/kg i.p. and 10 mg/kg i.p., respectively) (Wellington et al. 2013). Once adequate anesthesia was obtained, rats were subjected to laparotomy, and the left ureter was delicately isolated and tied with 4/0 surgical silk in two separate points at the ureteropelvic junction and the bladder, and then the ureter was surgically resected in between the two ligatures. Sham-operated animals were subjected to the same manipulations without ureter ligation. After the abdominal wound was stitched up using 3/0 surgical silk, the animals were allowed to heal in separate cages for 3 days (Hosseinian et al. 2017).

### Experimental design

Five groups of ten rats each (10 rats/group) were created randomly and distributed as follows: *Sham-operated group* underwent laparotomy without left ureter ligation and administered the vehicle, carboxy methyl cellulose (CMC, 0.5%) at a dose of (0.5 ml/100 g/day, orally) for 21 days. *UUO group* underwent left ureter ligation and administered the vehicle only at a dose of (0.5 ml/100 g/day, orally) for 21 days. *UUO + SOF group* underwent left ureter ligation and administered SOF (5 mg/kg/day, orally) (Ma et al. 2016) for 21 days. *UUO + EDV group* underwent left ureter ligation and administered EDV (20 mg/kg/day, orally) (Hassanein et al. 2021) for 21 days. *UUO + SOF + EDV group* underwent left ureter ligation and administered SOF (5 mg/kg/day, orally) and EDV (20 mg/kg/day, orally) for 21 days. The administration of doses for all groups was fixed from 12:00 PM to 2:00 PM every day until the end of the experiment.

At the end of the experiment, the animals underwent ketamine anesthesia (100 mg/kg i.p.) (Ali et al. 2021) followed by a cardiac puncture technique for blood sample collection. The serum was then separated after being centrifuged at 4000 ×g for 10 min at 4 °C and used to measure kidney function biomarkers. Left kidneys were dissected, rinsed with normal saline 0.9%, and weighed to calculate the renal somatic index (RSI). After that, the left kidneys were sliced into four pieces. For western blot analysis for protein quantification, the first piece was kept in RIPA buffer (Biospes, China) containing protease inhibitors at −80 °C. The second piece was stored in RNA latar® solution (Ambion, USA) at −80 °C for qRT-PCR investigation. To assess the oxidative stress and fibrotic biomarkers, the third piece was homogenized in ice-cold phosphate buffer saline (pH 7.4) to yield 10% homogenates. The final piece was preserved in a 10% neutral buffered formalin for histopathological and immunohistochemical investigations.

### Assessment of renal somatic index

After 24 h from the end of the experiment, the final body weight and left kidney of each rat were weighed, and RSI was calculated using the following equation (Abdel-Rahman et al. 2023):

$$RSI\:=\:Left\;kidney\;weight\;(g)/Final\;body\;weight\;of\;animal\;(g)\;\times\;100$$ 

### Assessment of renal oxidative stress biomarkers

#### Determination of reduced glutathione (GSH) content

The previously described method by Ellman (1959) was used to estimate the renal content of GSH. In brief, SH moiety reduces Ellman’s reagent to form 1 mol of 2-nitro-5-mercaptobenzoic acid per mole of SH. The resultant was measured spectrophotometrically at 412 nm.

#### Determination of lipid peroxides

Malondialdehyde (MDA) level in the kidney tissues was assessed to quantify lipid peroxidation following the method of Uchiyama and Mihara (1978). It was based on the interaction of two molecules of thiobarbituric acid and one molecule of MDA in the sample at low pH (2–3) and 95 °C for 45 min. The resultant color was determined spectrophotometrically at 535 nm and 520 nm.

#### Determination of superoxide dismutase (SOD) activity

Renal SOD activity was estimated using the method reported by Marklund (1985); in brief, SOD inhibits pyrogallol autoxidation. SOD activity in the sample is directly proportional to the degree of inhibition.

### Western blot analysis

Using ready-prepared protein extraction kits from Bio-Rad Laboratories Inc. California, USA, proteins were extracted from the rat’s left kidney. The total protein concentrations in tissue samples were assessed according to Bradford (1976). A comparable amount of total protein (30 µg) was transferred to a PVDF membrane (Millipore, Merck, Germany) after being separated on a 10% SDS-poly acrylamide gel according to the previously described (Towbin et al. 1979) wet transfer method. The membranes were blocked for 1 h at room temperature using 3% skim milk in Tris-buffered saline with Tween 20 (TBST). The membranes were then incubated overnight at 4 °C with the following primary antibodies, RIPK-1 (dilution; 1:500), MLKL (dilution; 1:500), RIPK-3 (dilution; 1:500), caspase-8 (dilution; 1:1000), and β-actin (dilution; 1:3000), followed by an incubation with secondary antibodies (linked to alkaline phosphatase) for 1 h. The protein bands were detected using a particular BCIP/NBT detection kit. Finally, protein levels were analyzed and compared using ImageJ® software (National Institutes of Health, Bethesda, USA).

### Immunohistochemistry

The procedures for immunological staining were carried out as described by Khalil et al. (2020). Deparaffinized renal sections were then dipped into a 0.05 M citrate buffer solution at a pH of 6.8 for antigen retrieval. After that, 0.3% H_2_O_2_ and protein block were applied to these sections. Then, the sections were incubated with the specific primary antibodies for MPO (dilution;1:200), Nrf2 (dilution;1:100), and HO-1 (dilution;1:100) overnight at 4 °C. They were then washed with phosphate-buffered saline solution and incubated with the secondary antibodies linked to (horseradish peroxidase) for 30 min at room temperature. The slides were initially visualized with a DAB kit, and then Mayer’s hematoxylin stain was used as a counterstain. Quantification of immunopositive expression of Nrf2 and HO-1 was conducted as area percent of six different fields using ImageJ® software (National Institutes of Health, Bethesda, USA) (Ramos-Vara and Miller 2014).

### Real-time (RT-qPCR)

For the entire RNA extraction process, ice-cold ingredients were used. In an RNase-free environment, an RNA extraction kit was used to extract pure total RNA from kidney tissue following the manufacturer’s instructions. RNA concentration and purity were assessed with the 260/280 ratio using a NanoDrop 2000 spectrophotometer. Following the producer’s instructions, RNA was reverse transcribed into complementary DNA (c-DNA) using the Revert-aid c-DNA synthesis kit (Thermo Scientific, USA). A real-time thermal cycler (Applied Biosystem StepOnePlus, USA) was then used to amplify the complementary DNA. The amplification reaction mixture (15 µl) contains 2 µl of complementary DNA, 0.5 µl of each primer, 7.5 µl of SYBR Green universal master mix (Thermo Scientific, USA), and 4.5 µl of nuclease-free water. According to Livak and Schmittgen (2001), the comparative cycle threshold (Ct) ( 2^−△△Ct^) was used to analyze the resulting data and calculate the relative expression. Expression levels were normalized to GAPDH. The following primers were used: TNF-α: 5′-CAGCCTTGTCCCTTGAAGAGAACC-3′ (forward), 5′-TACTGAACTTCGGGGTGATTGGTCC-3′ (reverse); collagen-1α: 5′-GAAACCCGAGGTATGCTTGA-3′ (forward), 5′-GACCAGGAGGACCAGGAAGT-3′ (reverse); GAPDH: 5′-TGCTGGTGCTGAGTATGTCG-3′ (forward), 5′-TTGAGAGCAATGCCAGCC-3′ (reverse).

### Estimation of renal IL-1β, HYP, and TNF-α expression level

IL-1β, HYP, and TNF-α levels in renal tissue were estimated using the ELISA technique based on the manufacturer’s protocol and following the earlier reported procedures (Van Weemen and Schuurs 1971).

### Histopathological analysis

Samples of kidney tissue were fixed in 10% neutral buffered formalin for 72 h. The tissues were dehydrated and cleared before being fixed in paraffin and sectioned into 5 μm sections. The serial sections were stained for histological investigation using hematoxylin and eosin (H&E) and Masson’s trichrome stains in accordance with the previously reported protocol (Suvarna et al. 2018). According to Geleilete et al. (2001), each group’s histopathological changes in kidney tissues were graded on a scale of 0 to 4. Masson’s trichrome stain was applied to assess the extent of collagen fiber deposition. Using the ImageJ® (National Institutes of Health, Bethesda, USA), ten fields (area %, the proportion of the stained area compared to the overall area) were used to measure the fibrotic areas. The Olympus® BX51 optical microscope (Olympus Corporation, Tokyo, Japan) was used to capture the images. The histopathological investigations were done by an expert pathologist unaware of the group’s allocation.

### Statistical analysis

Data are shown as means ± standard error of the means (SEM). The statistical variations were examined using one-way analysis of variance (ANOVA) for parametric data followed by Tukey-Kramer as a post hoc test. On the other hand, the Kruskal-Wallis test for non-parametric data. Data analyses were performed by the GraphPad Prism software program (V. 7). At *P* < 0.05, statistical significance was considered acceptable.

## Results

### Effects of SOF, EDV, and their combination on the mass index and morphology of the left kidney

**Fig. 1:**
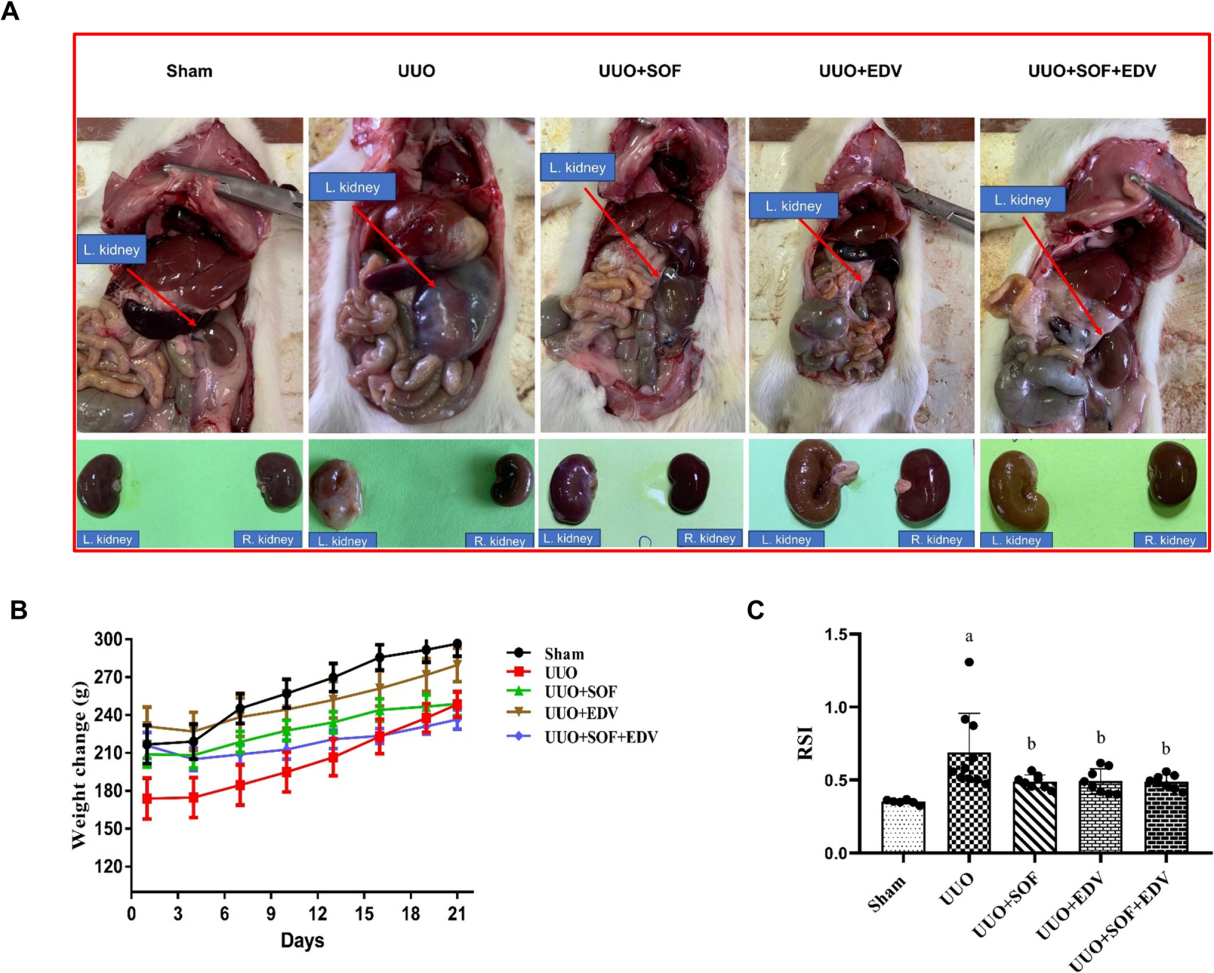
Effect of SOF, EDV, and their combination on the left kidney morphology and RSI. **A** Left kidney appearance. **B** Animals’ weight change. **C** RSI. Data are shown as mean ± standard error of the mean (*n* = 8). Multiple comparisons were performed using analysis of variance test followed by Tukey-Kramer as post hoc test; a: versus sham control group; b: versus UUO group; c: versus UUO + SOF + EDV group at *P*-value < 0.05

During the macroscopic examination, the left kidneys from the UUO group appeared larger, paler in color, and more fibrotic than those from the sham-operated group. On the other hand, pretreatment with SOF, EDV, and their combination restored these macroscopic features. The treated kidneys appeared smaller, nearly normal color, and much less fibrotic than those of the UUO rats (Fig. 1A). A steady increase in the animals’ weight in all groups was observed with no significant change (Fig. 1B). Additionally, in UUO rats, RSI increased by almost twofold. However, pretreatment with SOF, EDV, and their combination reduced these elevations in RSI by around 1.4-, 1.4-, and 1.3-fold, respectively at *P* < 0.05 (Fig. 1C).

### Effects of SOF, EDV, and their combination on kidney function and serum electrolyte biomarkers

**Fig. 2:**
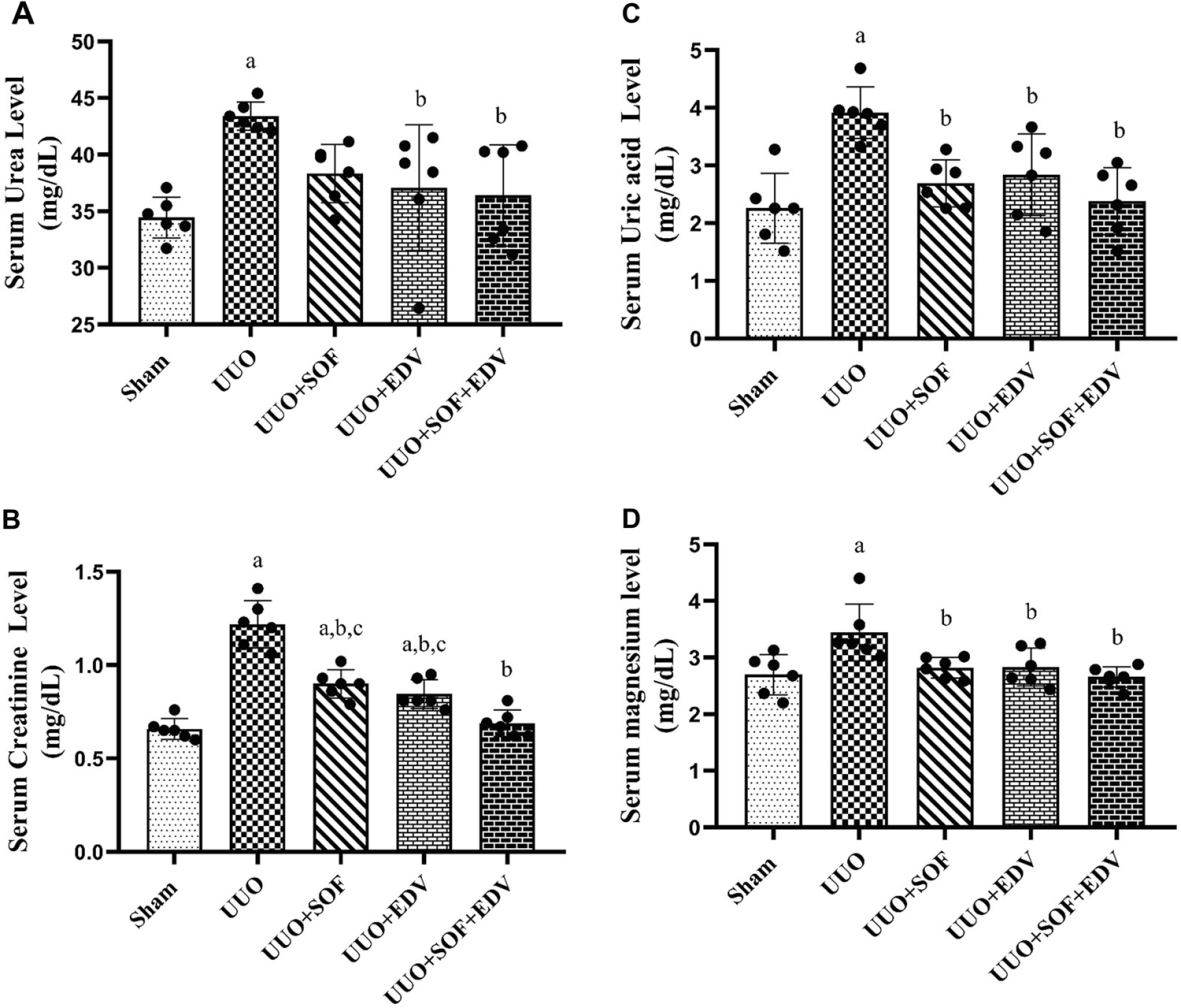
Effect of SOF, EDV, and their combination on serum urea, creatinine, uric acid, and magnesium levels. **A** Serum urea level. **B** Serum creatinine level. **C** Serum uric acid level. **D** Serum magnesium level. Data are shown as mean ± standard error of the mean (*n* = 6). Multiple comparisons were performed using analysis of variance test followed by Tukey-Kramer as post-hoc test; a: versus sham control group; b: versus UUO group; c: versus UUO + SOF + EDV group at *P*-value < 0.05

As demonstrated in (Fig. 2A, B, C, and D), UUO-exposed rats showed a marked elevation of serum urea, creatinine, uric acid, and magnesium by 1.3-, 1.8-, 1.7-, and 1.3-fold, respectively, compared to the sham-operated rats at *P* < 0.05. In contrast, pretreatment with SOF, EDV, and their combination restored the levels of those renal biomarkers to normal or near-normal values.

### Effects of SOF, EDV, and their combination on histopathological changes induced by UUO

**Fig. 3:**
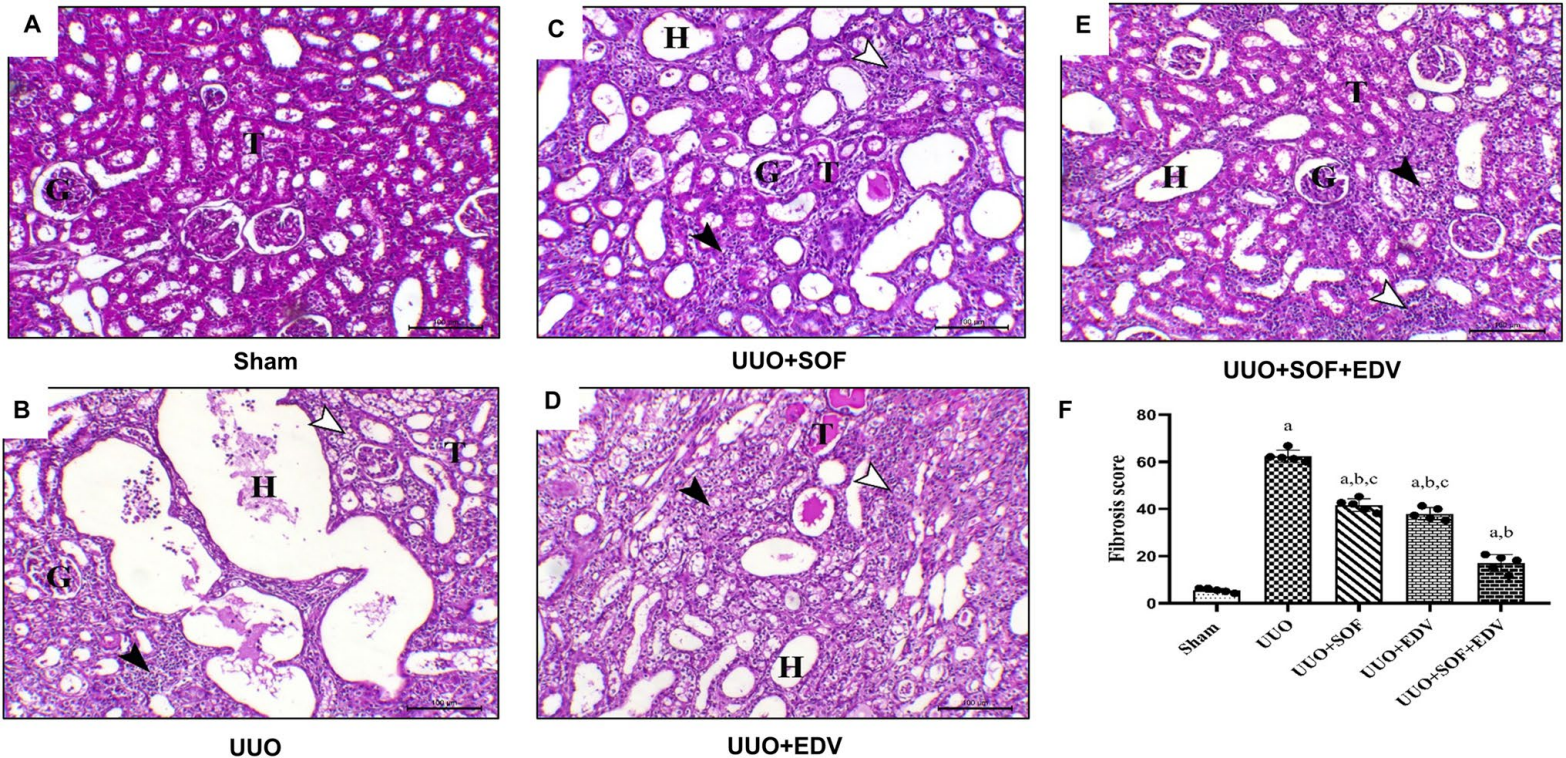
Effects of SOF, EDV, and their combination on histopathological changes induced by UUO. Photomicrographs of kidney sections stained by hematoxylin and eosin (100×). **A** Kidney section of sham animal showing normal renal glomeruli and tubules (G and T, respectively). **B** Kidney section of UUO animal showing marked hydronephrosis (H), interstitial nephritis associated with mononuclear inflammatory cells infiltration (black arrowhead), tubular degeneration (white arrowhead), and interstitial fibrosis (G, T, and H letters indicate renal glomeruli and tubules and hydronephrosis respectively). **C** Kidney section of UUO animal treated with SOF showing decreased lesions associated with UUO model and the renal tissues revealing moderate hydronephrosis (H) and decreased both interstitial nephritis (black arrowhead) and tubular degeneration (white arrowhead) (G, T, and H letters indicate renal glomeruli and tubules and hydronephrosis respectively). **D** Kidney section of UUO animal treated with EDV showing moderate hydronephrosis (H), decreased interstitial nephritis (white arrowhead), and tubular degeneration associated with protein secretion within the lumen of the renal tubules (black arrowhead) (G, T, and H letters indicate renal glomeruli and tubules and hydronephrosis, respectively). **E** Kidney of UUO animal treated with the combination of SOF and EDV showing a marked decrease in the lesions of UUO that demonstrated mild hydronephrosis (H), slight interstitial nephritis (white arrowhead) and mild tubular degeneration (black arrowhead) (G, T, and H indicate renal glomeruli and tubules and hydronephrosis, respectively). Scale bar = 100 μm (for 100× images). **F** Semi-quantitative fibrosis score. Data are expressed as mean ± Standard error of the mean (*n* = 5). Multiple comparisons were conducted using the Kruskal-Wallis test; a: versus sham control group; b: versus UUO group; c: versus UUO + SOF + EDV group at *P*-value < 0.05

Histopathological examination by routine H&E stain was employed to evaluate the possible protective effect of EDV, SOF, and their combination against UUO-induced tissue injury. As illustrated in Fig. 3, the sham control group showed normal renal glomeruli and tubules (Fig. 3A). On the other hand, animals subjected to the UUO challenge showed marked hydronephrosis, interstitial nephritis associated with mononuclear inflammatory cell infiltration, tubular degeneration, and interstitial fibrosis (Fig. 3B). UUO kidneys pretreated with SOF showed decreased lesions as indicated by moderate hydronephrosis and decreased interstitial nephritis and tubular degeneration (Fig. 3C). Additionally, UUO kidneys pretreated with EDV showed mild hydronephrosis, reduced interstitial nephritis, and tubular degeneration associated with protein secretion within the lumen of the renal tubules (Fig. 3D). Finally, kidneys of UUO animals pretreated with the combination of SOF and EDV showed a marked decrease in the lesions of UUO that demonstrated mild hydronephrosis, slight interstitial nephritis, and mild tubular degeneration (Fig. 3E).

### Effects of SOF, EDV, and their combination on fibrotic markers

**Fig. 4:**
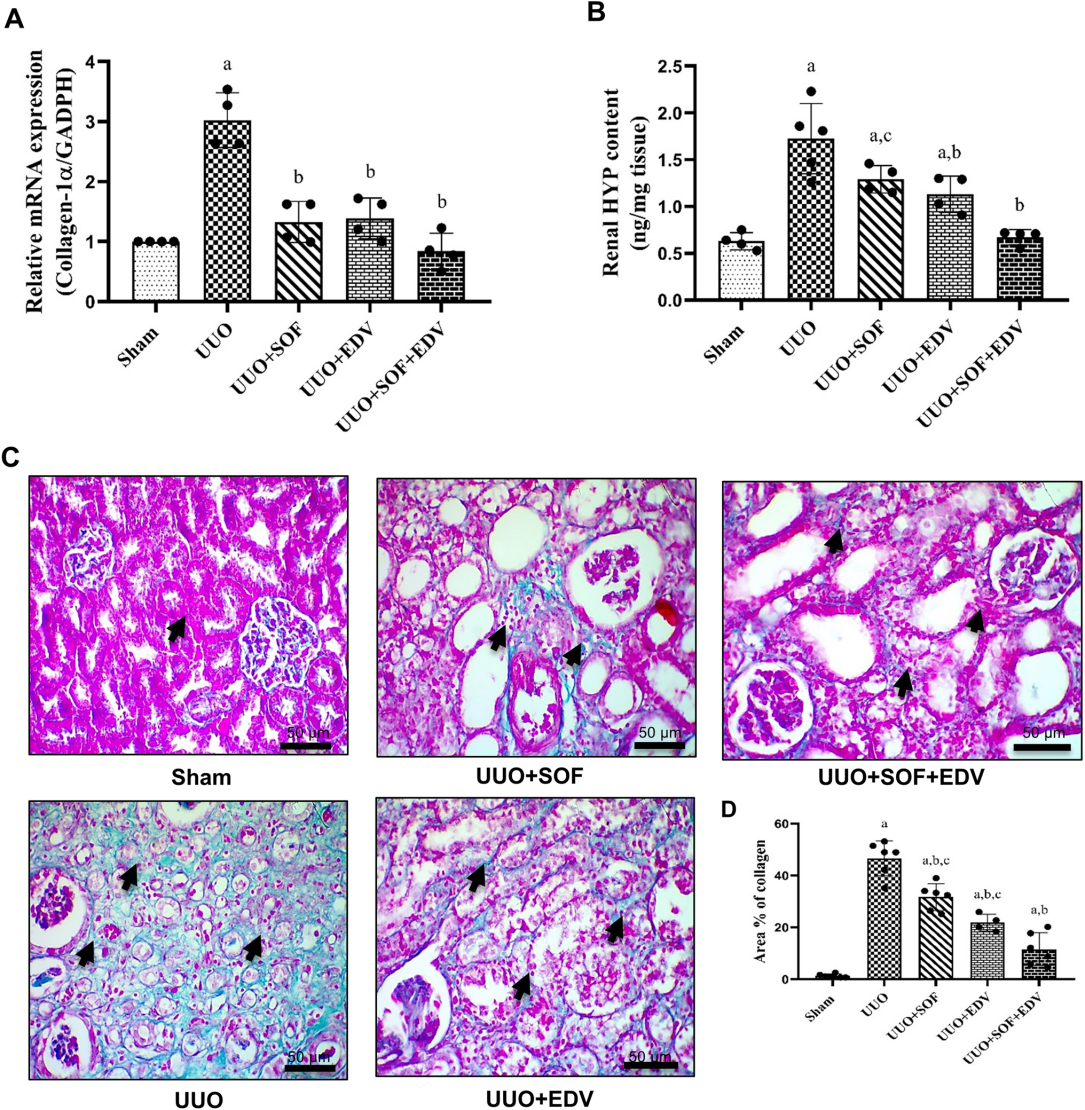
Effects of SOF, EDV, and their combination on mRNA expression of collagen-1α and renal HYP content as well as collagen deposition. **A** Total RNA was extracted from the kidney tissues of sham, UUO, UUO + SOF, UUO + EDV, and UUO + SOF + EDV groups. The mRNA transcription of collagen-1α was detected using real-time PCR analysis. **B** Tissue levels of HYP of sham, UUO, UUO + SOF, UUO + EDV, and UUO + SOF + EDV groups were determined by ELISA. **C** To assess the extent of collagen fiber deposition, Masson trichrome staining of left kidney sections obtained from the kidney of sham, UUO, UUO + SOF, UUO + EDV, and UUO + SOF + EDV groups was performed. Scale bar = 50 μm (for 200× images). **D** Area % of collagen deposition. Data are shown as mean ± standard error of the mean (*n* = 6). Multiple comparisons were conducted using a one-way analysis of variance followed by Tukey-Kramer as post-ANOVA test for parametric data and Kruskal-Wallis test for non-parametric data; a: versus sham control group; b: versus UUO group; c: versus UUO + SOF + EDV group at *P*-value < 0.05

Furthermore, the degree of fibrosis was confirmed by assessing collagen-1α and HYP expressions as well as tissue collagen deposition. The mRNA expression of collagen-1α was significantly increased in the UUO group by threefold compared to the sham-operated group (Fig. 4A). In contrast, administration of SOF, EDV, and their combination significantly downregulated the mRNA expression of collagen-1α by 56%, 54%, and 72%, respectively, compared to the UUO group. In addition, protein expression of HYP was markedly increased in the UUO group by 2.7-fold as compared to the sham control group (Fig. 4B). Conversely, pretreatment with SOF, EDV, and their combination showed a downregulation of HYP expressions by 25%, 34%, 61%, respectively, compared to UUO rats. For further confirmation, collagen deposition was investigated by Masson trichrome staining. As shown in Fig. 4C, the UUO group showed marked interstitial fibrosis and collagen deposition 35-fold as compared to the sham-operated group. On the contrary, pretreatment with SOF, EDV, and their combination significantly decreased renal tubulointerstitial damage and collagen deposition by 32%, 53%, and 75%, respectively at *P* < 0.05.

### Effects of SOF, EDV, and their combination on renal oxidative stress biomarkers

**Fig. 5:**
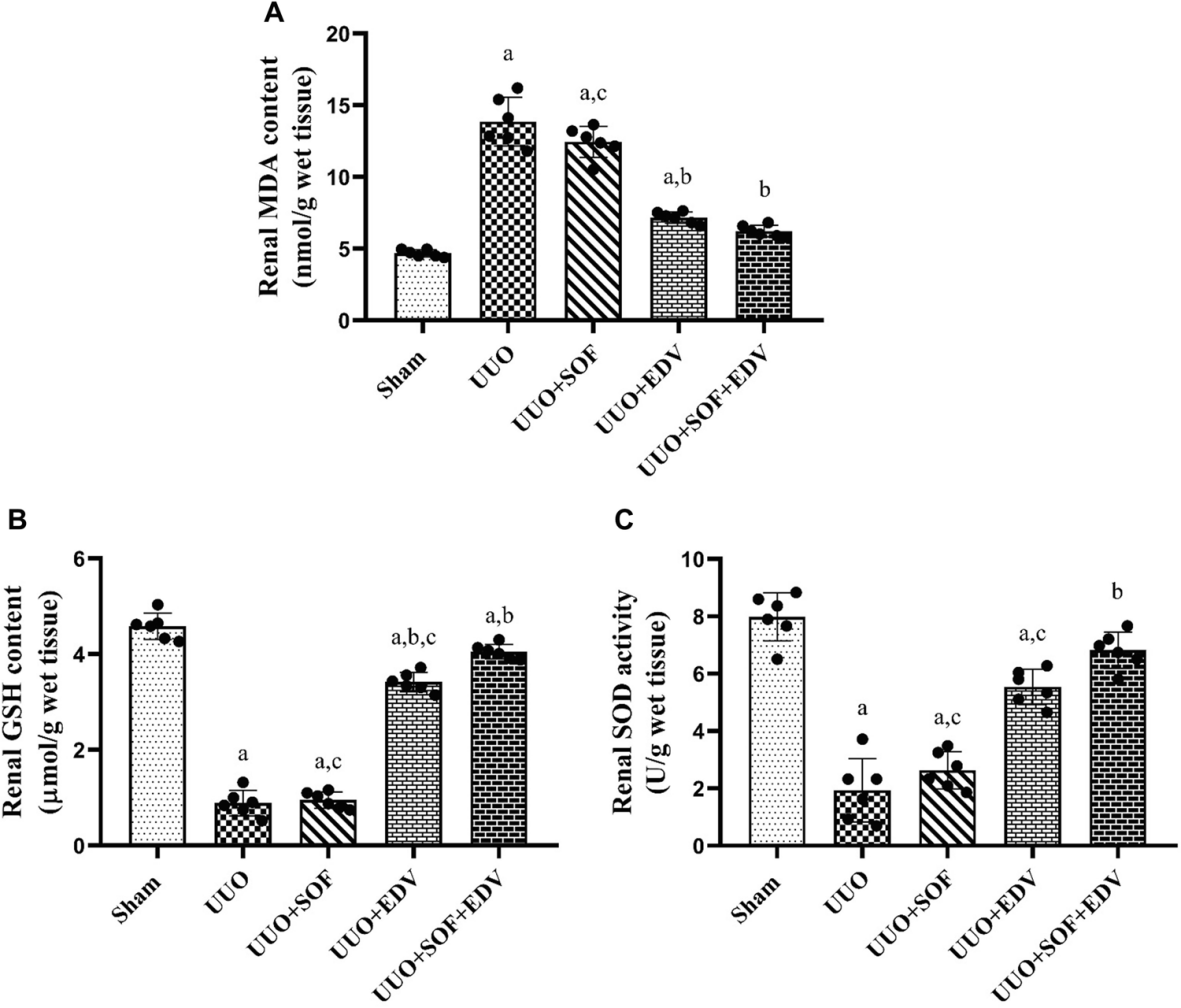
Effects of SOF, EDV, and their combination on renal MDA and GSH contents as well as SOD activity. **A** Malondialdehyde. **B** Reduced glutathione. **C** Superoxide dismutase. Data are shown as mean ± standard error of the mean (*n* = 6). Multiple comparisons were performed using analysis of variance test followed by Tukey-Kramer as post hoc test; a: versus sham control group; b: versus UUO group; c: versus UUO + SOF + EDV group at *P*-value < 0.05

As illustrated in Fig. 5, UUO induced marked elevation in renal MDA content by 2.9-fold, whereas GSH content and SOD activity were markedly reduced by 80% and 75%, respectively, compared to the sham-operated group. On the other hand, administration of SOF slightly elevated renal GSH content and SOD activity by 1.1-fold and 1.3-fold, respectively (Fig. 5B and C) and reduced MDA level by 10% compared to UUO rats (Fig. 5A). While administration of EDV markedly elevated renal GSH content and SOD activity by 3.8-fold and 2.8-fold, respectively (Fig. 5B and C) and diminished MDA level by 48% compared to UUO rats (Fig. 5A). Interestingly, pretreatment with SOF and EDV in combination significantly elevated renal GSH content and SOD activity by 4.5-fold and 3.5-fold, respectively, and diminished MDA level by 55% compared to the UUO group.

### Effects of SOF, EDV, and their combination on renal Nrf2 and HO-1 expression

**Fig. 6:**
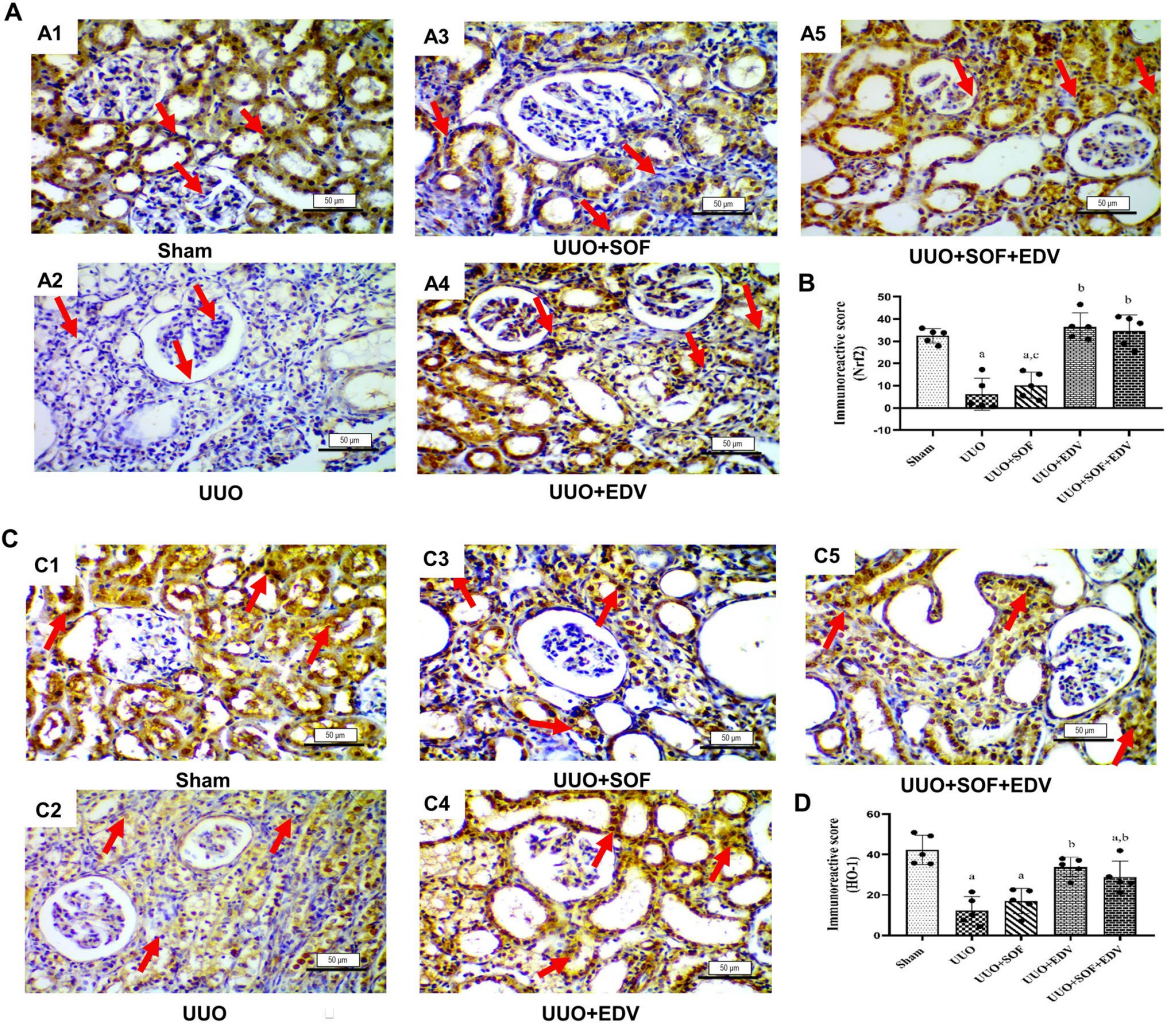
Effects of SOF, EDV, and their combination on renal Nrf2 and HO-1 expression. Immunohistochemical staining of Nrf2 (**A**) and HO-1 (**C**) in kidneys of sham, UUO, UUO + SOF, UUO + EDV, and UUO + SOF + EDV groups (X200). (A1) Nrf2 is mildly expressed in the kidney of the sham group. (A2) Kidneys of UUO animals showing significant reduction in Nrf2 expression. (A3) Kidneys of UUO animals treated with SOF showed no significant change in the expression of Nrf2. (A4) Kidneys of UUO animals treated with EDV showed significant elevation in Nrf2 expression. (A5) Kidneys of UUO animals treated with a combination of SOF + EDV showed a marked increase in the expression of Nrf2. Scale bar = 50 μm. **B** Semi-quantitative scores of renal Nrf2 expression. (C1) HO-1 is mildly expressed in the kidney of the sham group. (C2) Kidneys of UUO animals showing significant reduction in HO-1 expression. (C3) Kidneys of UUO animals treated with SOF showed no significant change in the expression of HO-1. (C4) Kidneys of UUO animals treated with EDV showed significant elevation in HO-1 expression. (C5) Kidneys of UUO animals treated with a combination of SOF + EDV showed a marked increase in the expression of HO-1. Scale bar = 50 μm. **D** Semi-quantitative scores of renal HO-1 expressions. Data are expressed as mean ± standard error of the mean (*n* = 5). Multiple comparisons were performed using the Kruskal-Wallis test; a: versus sham control group; b: versus UUO group; c: versus UUO + SOF + EDV group at *P*-value < 0.05

Immunohistochemistry revealed that UUO-ligated kidneys showed a marked decrease in Nrf2 (Fig. 6A) and HO-1 (Fig. 6C) expression by 81% and 71%, respectively, compared to sham rats. In contrast, EDV administration significantly increased renal Nrf2 (Fig. 6A4) and HO-1 (Fig. 6C4) expression by 5.9-fold and 2.8-fold, respectively, than their level in UUO rats. At the same time, SOF failed to produce a significant change in their expression (Fig. 6A3, C3). In contrast, the combination of EDV with SOF exhibited a significant elevation of Nrf2 (Fig. 6A5) and HO-1 (Fig. 6C5) expression levels by 5.6-fold and 2.4-fold, respectively, compared to UUO rats.

### Effects of SOF, EDV, and their combination on TNF-α and IL-1β expression as well as MPO activity

**Fig. 7:**
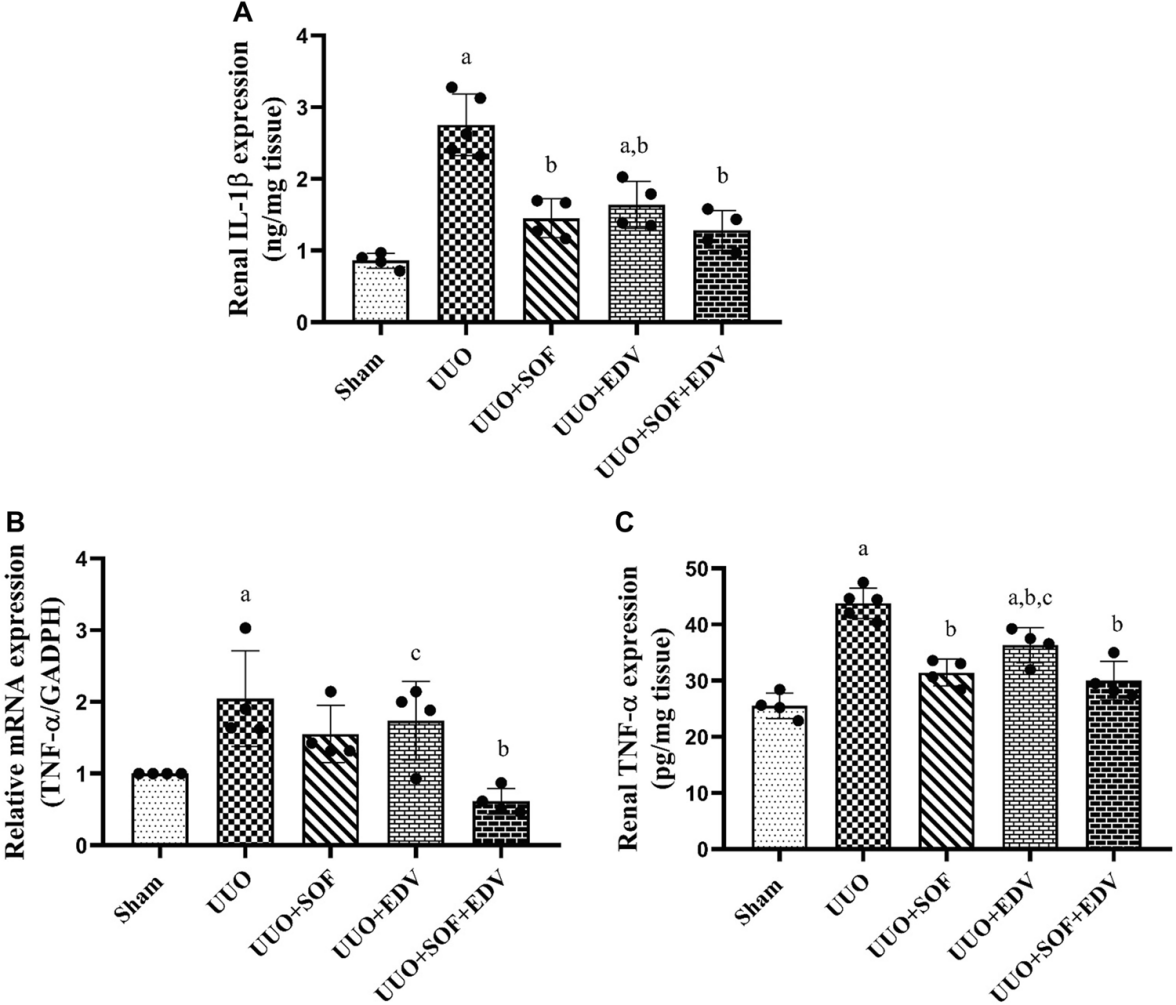
Effects of SOF, EDV, and their combination on IL-1β and TNF-α expression. The levels of either IL-1β (**A**) or TNF-a (**C**) in the kidneys of sham, UUO, UUO + SOF, UUO + EDV, and UUO + SOF + EDV groups were determined by ELISA. From the kidney tissues of sham, UUO, UUO + SOF, UUO + EDV, and UUO + SOF + EDV groups, total RNA was extracted. The mRNA transcription of TNF-α (**B**) was detected using real-time PCR analysis. The mean fold induction of TNF-a’s mRNA, which was normalized to GAPDH, is displayed. Data are expressed as mean ± standard error of the mean (*n* = 4). Multiple comparisons were conducted using a one-way analysis of variance followed by Tukey-Kramer as a post hoc test for parametric data and Kruskal-Wallis test for non-parametric data; a: versus sham control group; b: versus UUO group; c: versus UUO + SOF + EDV group at *P*-value < 0.05

Here, renal proinflammatory cytokine IL-1β expression markedly increased in UUO rats 3.2-fold compared to the sham-operated group (Fig. 7A). In comparison, mRNA and protein expression levels of TNF-α were significantly increased in UUO rats by twofold and 1.7-fold, respectively (Fig. 7B and C). On the contrary, administration of SOF, EDV, and their combination significantly inhibited the protein expression of IL-1β by 47%, 40%, and 53%, respectively, compared to UUO rats. Moreover, compared to UUO animals, pretreatment with SOF, EDV, and their combination markedly decreased the levels of TNF-α on mRNA levels by 24%, 15%, and 70%, as well as on protein levels by 28%, 17%, and 31%, respectively, at *P* < 0.05.

**Fig. 8:**
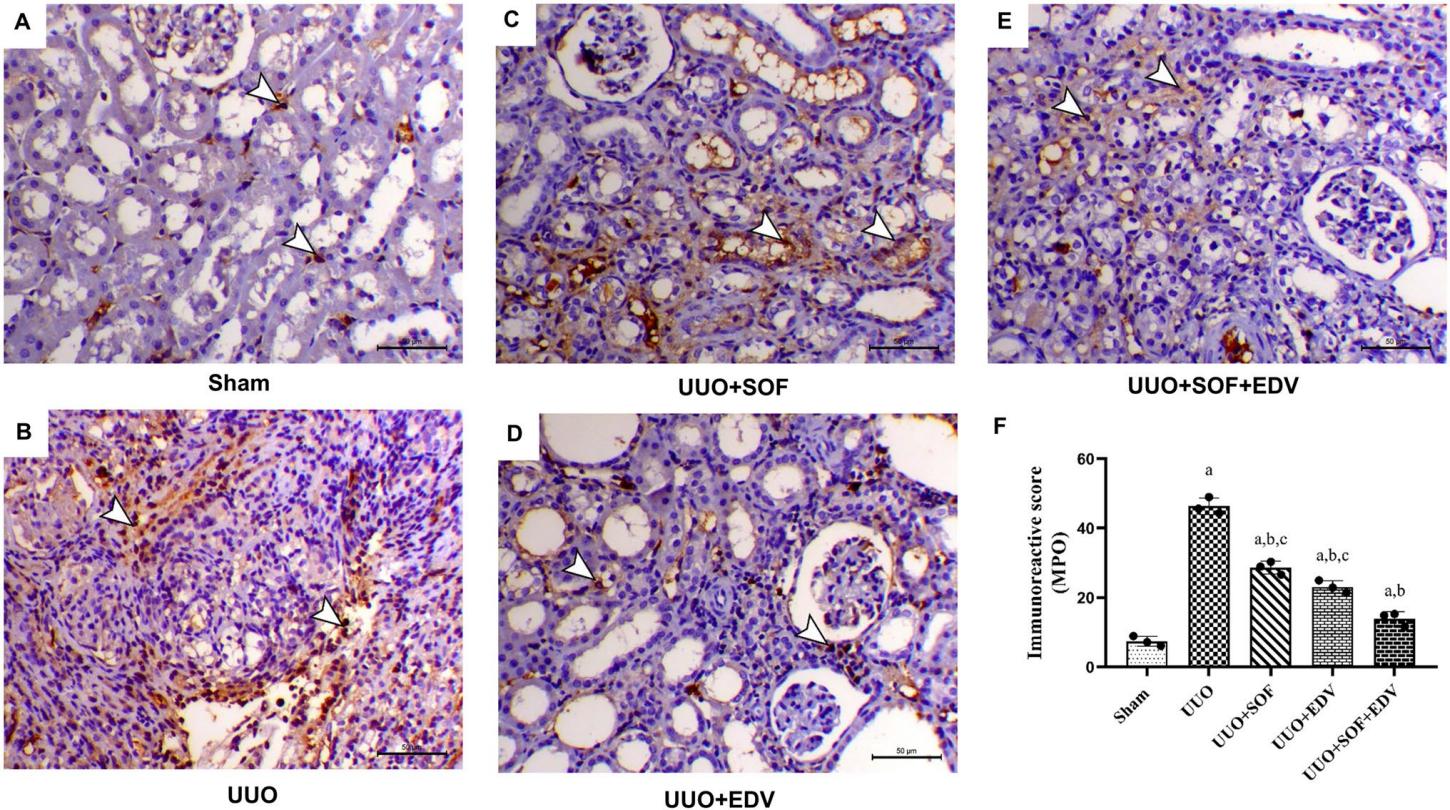
Effects of SOF, EDV, and their combination on MPO expression. Immunohistochemical staining of MPO in kidneys of sham, UUO, UUO + SOF, UUO + EDV, and UUO + SOF + EDV groups (×200). **A** Kidney of the sham group showing mild expression of MPO unless slight immunostaining within the interstitial cells (arrowheads). **B** Kidney of UUO animal showing marked expression of MPO, especially within the interstitial inflammatory cells (arrowheads). **C** Kidney of UUO animals treated with SOF showed a decrease in the expression of MPO especially within the tubular renal cells (arrowheads). **D** Kidney of UUO animals treated with EDV showing a decrease in the expression of MPO especially within the interstitial inflammatory cells (arrowheads). **E** Kidney of UUO animal treated with a combination of SOF and EDV showing a marked decrease in the expression of MPO within the interstitial inflammatory cells (arrowheads); scale bar = 50 μm. **F** Semi-quantitative scores of renal MPO expression. Data are expressed as mean ± standard error of the mean (*n* = 3). Multiple comparisons were conducted using a one-way analysis of variance followed by Tukey-Kramer as a post hoc test for parametric data and Kruskal-Wallis test for non-parametric data; a: versus sham control group; b: versus UUO group; c: versus UUO + SOF + EDV group at *P*-value < 0.05

As demonstrated in Fig. 8, the renal section of sham-operated rats showed mild MPO expression, particularly in the interstitial cells, whereas kidneys of UUO rats exhibited marked expression of MPO, especially within the interstitial inflammatory cells, by 6.2-fold compared to the sham-operated group (Fig. 8B). In contrast, in SOF-pretreated rats, MPO expression decreased within the interstitial inflammatory cells and tubular renal cells by 38% compared to the UUO group (Fig. 8C). Moreover, pretreatment with EDV showed reduced MPO expression, especially within the interstitial inflammatory cells, by 50% more than that of UUO rats (Fig. 8D). Interestingly, combination therapy shows a marked decrease in MPO expression within the interstitial inflammatory cells and tubular epithelium by 70% compared to UUO kidneys (Fig. 8E).

### Effects of SOF, EDV, and their combination on RIPK-1, RIPK-3, MLKL, and caspase-8 expression

**Fig. 9:**
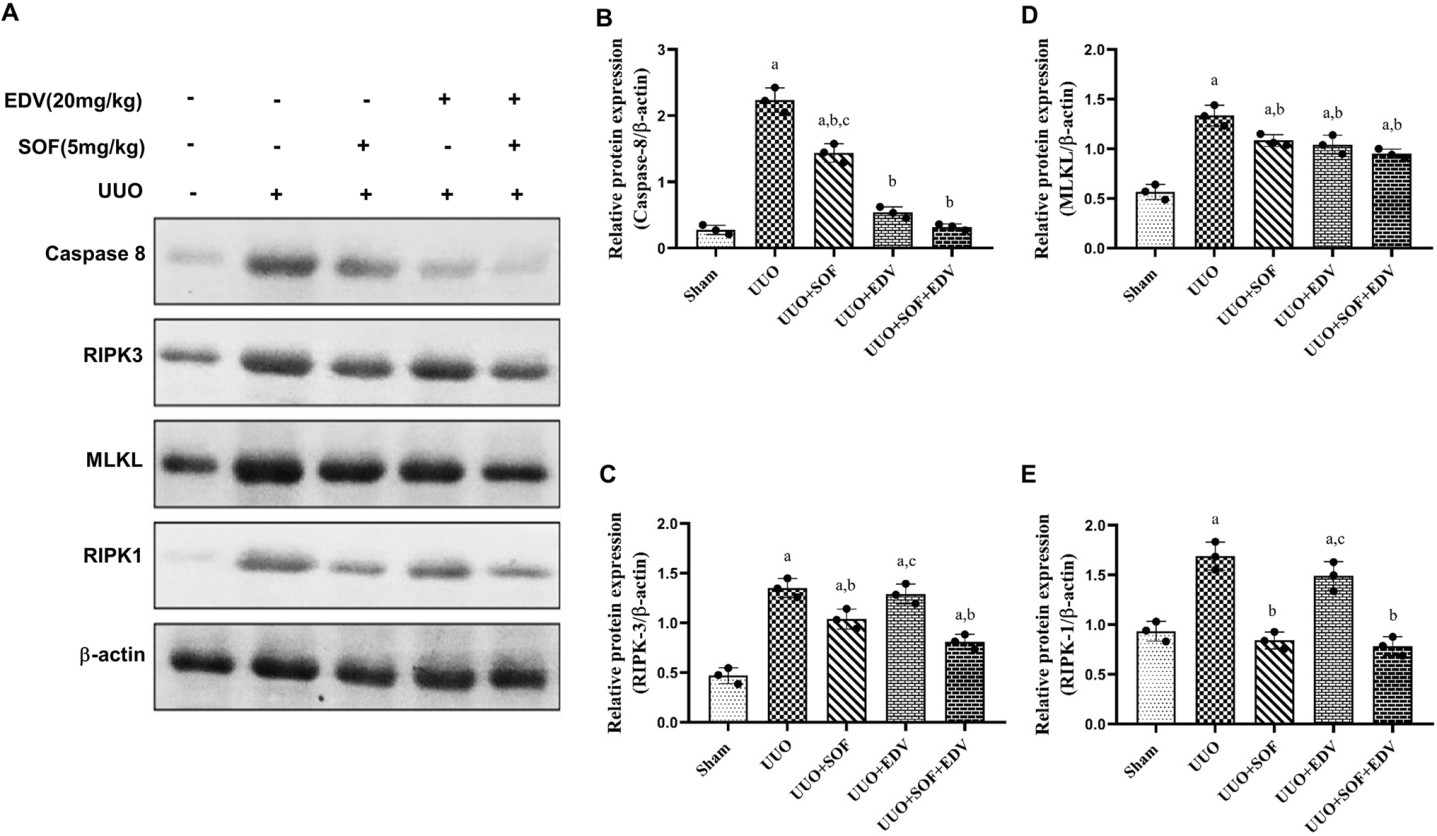
Effects of SOF, EDV, and their combination on protein expression of caspase-8, RIPK-3, MLKL and RIPK-1. Total extracts from the kidneys of sham, UUO, UUO + SOF, UUO + EDV, and UUO + SOF + EDV groups were submitted to western blot analysis and probed with the antibodies (**A**). The right panels show caspase 8 (**B**) and RIPK-3 (**C**), MLKL (**D**), and RIPK-1 (**E**) densitometric analysis in relation to the b-actin level. Data are shown as mean ± standard error of the mean (*n* = 3). Multiple comparisons were performed using analysis of variance test followed by Tukey-Kramer as post hoc test; a: versus sham control group; b: versus UUO group; c: versus UUO + SOF + EDV group at *P*-value < 0.05

To assess the examined drugs’ anti-fibrotic effect, the protein expression of RIPK-1, RIPK-3, MLKL, and caspase-8 which are implicated in the necroptotic pathway was investigated. It was found that UUO caused a significant upregulation of all of these proteins by 1.8-, 2.9-, 2.4-, and eightfold, respectively, as compared to sham-operated rats. On the contrary, administration of SOF significantly reduced RIPK-1, RIPK-3, MLKL, and caspase-8 expression by 50%, 23%, 19%, and 36%, respectively, as compared to the UUO group, while administration of EDV significantly reduced RIPK-1, RIPK-3, MLKL, and caspase-8 expression by 12%, 5%, 22%, and 76%, respectively, compared to the UUO group. Moreover, the combination of SOF and EDV significantly reduced RIPK-1, RIPK-3, MLKL, and caspase-8 expression by 54%, 40%, 29%, and 86%, respectively, compared to the UUO group at *P* < 0.05 as shown in (Fig. 9A–E).

## Discussion

Chronic obstructive nephropathy in humans is believed to be similar to experimental UUO in rodents (Martínez-Klimova et al. 2019). So, in this study, we tried to elucidate the potential anti-fibrotic effects of SOF, EDV, and their combination using the UUO model. Furthermore, the molecular mechanisms by which these drugs exert the anti-fibrotic effects were also investigated. The present study demonstrates that UUO causes renal fibrosis and a decline in renal function as indicated by a significant rise in serum levels of creatinine, uric acid, urea, and magnesium in the UUO group. These results are consistent with previous studies (Zhao and Luan 2020; Hassan et al. 2021). Pretreatment with SOF, EDV, and their combination considerably attenuated these changes in renal function markers, suggesting that it could diminish renal damage and improve the functionality of obstructed kidneys.

Reactive oxygen species (ROS), secondary messengers, are important for active solute transport and reabsorption. Excessive ROS build-up causes renal fibrosis and is connected to several kidney diseases. As a result, treating renal fibrosis by lowering ROS generation could be effective (Liang et al. 2014). Increased levels of ROS can damage the tubulointerstitial tissues through accelerating lipid peroxidation, leukocyte activation, hydrogen peroxide production, protein oxidation, DNA oxidation, and apoptosis. Renal fibrosis progresses as a result of initial oxidative damage and inflammation. Consequently, kidney damage caused by UUO has been prevented by using antioxidant and/or anti-inflammatory medications (Li et al. 2017). Recently, multiple studies revealed that reducing the production of ROS may be crucial in treating and preventing renal fibrosis associated with unilateral ureter obstruction (Ram et al. 2022; Liang et al. 2014). Our findings demonstrated that compared to the sham-operated rats, left kidney tissues from UUO animals had significantly higher levels of MDA, a marker of lipid peroxidation, and considerably lower levels of the antioxidants SOD and GSH. Contrarily, pretreatment with EDV alone or in combination with SOF significantly lowered MDA levels and increased SOD and GSH activity, whereas SOF alone failed to produce a significant change in those markers. Similar findings were reported by Liu et al. (2015).

It has been demonstrated that the cytoprotective function of Nrf2 is essential for the tissues that are subjected to oxidative stress (Mahmoud et al. 2017; Mahmoud and Al Dera 2015). Thus, it has been found that Nrf2 was a crucial molecular target for an array of cytoprotective and chemo-preventive drugs. In the present work, unilateral ureter obstruction exhibited a significant decrease in Nrf2/HO-1 expression and a subsequent reduction in antioxidants, which could be attributed to the harmful effects of UUO. On the other hand, pretreatment with EDV alone or in combination with SOF prevented the downregulation of Nrf2/HO-1 in the renal tissues of rats compared to UUO group; this result is consistent with the increased antioxidant enzyme levels. In rodent models of cerebral ischemia (Xu et al. 2022), traumatic brain injury (Zhang et al. 2019), and chlorpyrifos neurotoxicity (Shou et al. 2019), enhanced Nrf2 expression provides support for the idea that Nrf2 signaling may be involved in the beneficial effects of EDV. Therefore, one of the crucial mechanisms underlying the protective effect of EDV against UUO-induced renal fibrosis is Nrf2 activation.

Inflammation is a key contributor to UUO-induced renal fibrosis (Zheng et al. 2019; Kim et al. 2016). In the current work, the UUO group showed marked elevation in renal TNF-α and IL-1β proinflammatory cytokines compared to the sham-operated group. Contrarily, the expression of both TNF-α and IL-1β was significantly reduced in SOF, EDV, and their combination groups. These findings may point to the possibility that SOF and EDV have anti-inflammatory properties that could contribute to their anti-fibrotic effects. In line with these findings, EDV decreased the expression of TNF-α and IL-1β in systemic sclerosis (Yoshizaki et al. 2011) and cerebral ischemia-reperfusion injury (Liu et al. 2022). SOF also significantly diminished the serum level of TNF-α and IL-1β in rheumatoid arthritis (Wang et al. 2018b), and cisplatin-induced nephrotoxicity (Demirtas et al. 2023). Moreover, MPO activity could be used as a sign of tissue leukocyte buildup. The levels of MPO in tissue have frequently been used as a proxy for neutrophil infiltration (Demirbilek et al. 2007). In the present work, UUO renal tissues showed a marked elevation in the expression of MPO compared to sham-operated kidneys, whereas UUO-pretreated kidneys with SOF, EDV, and their combination exhibited a significant downregulation in MPO expression. In agreement with our data, previous studies showed that EDV can downregulate MPO expression in various animal models, including cerebral ischemia-reperfusion injury (Liu et al. 2022) and testicular damage (Hassanein et al. 2023). These findings suggested that SOF and EDV might have anti-inflammatory properties.

To further explore whether the examined drugs have an anti-fibrotic effect or not, the expression of HYP and collagen-1α in kidney tissues was measured. Renal fibrosis, which is thought to be a substantial pathogenic component of chronic renal disorders, is characterized by the excessive and persistent deposition of ECM (Nogueira et al. 2017). Overproduction of ECM, which results from an imbalance between ECM production and breakdown, is a critical component of the pathophysiology of renal fibrosis (Wang et al. 2017). Collagen 1 and other interstitial collagens are the main ECM components in interstitial fibrosis (Bülow and Boor 2019). Changes in the kidney’s HYP concentration are considered an indicator of collagen metabolism and can reveal important details about the pathophysiology of renal fibrosis. Accordingly, our results indicated that UUO exhibited a significant upregulation in HYP and collagen 1 protein expression than in the sham control group. On the other hand, pretreatment with SOF, EDV, and their combination significantly downregulated HYP and collagen 1 expression. These results indicated that SOF, EDV, and their combination could mitigate the pathogenesis of UUO-induced kidney fibrosis. In agreement with our results, the anti-fibrotic activity of SOF and EDV has been previously reported in the kidney (Ma et al. 2016), liver (Yuan et al. 2022), and lung (Wang et al. 2018a; Zhi et al. 2011). Furthermore, histological results validated the anti-fibrotic properties of SOF, EDV, and their combination. These findings suggest that the anti-fibrotic activity of these drugs could be responsible for their possible protective effect.

Consistently with the previous studies, UUO-induced inflammation and fibrosis are reduced by pharmacological inhibition of necroptosis or RIPK-3 genetic deficiency (Xiao et al. 2017; Imamura et al. 2018). In the current work, UUO significantly increased the protein expression of RIPK-1, RIPK-3, and MLKL compared to sham-operated rats. These data are in line with a previous study (Xiao et al. 2017). In contrast, administration of SOF significantly downregulated RIPK-1-RIPK-3 and MLKL expression. This is in agreement with a previous study (Martens et al. 2017; Zhao et al. 2022; Han et al. 2018). Additionally, the upregulated expression of caspase-8 in the UUO group was significantly decreased by administration of either SOF or EDV or both of them, indicating the anti-apoptotic effects of these drugs. This agrees with previous studies (Hong et al. 2013; Koike et al. 2021). These findings imply that the anti-fibrotic and anti-apoptotic properties of SOF and EDV may be responsible for their possible protective impact against UUO-induced renal fibrosis.

## Conclusions

This study established the potential involvement of the RIPK-3/MLKL necroptotic pathway, oxidative stress, and inflammation in the pathogenesis of renal fibrosis during the UUO model, whereas pretreatment of UUO rats with SOF and/or EDV attenuated renal impairments and ameliorated renal oxidative damage, inflammation, reduced tissue injury, and fibrosis. Moreover, SOF impact could be attributed to RIPK-3/MLKL necroptotic pathway suppression, and EDV exerts its activity by inhibiting oxidative stress and inflammation. Additionally, EDV greatly increased the sorafenib’s anti-fibrotic action, demonstrating that combination therapy is superior to using each drug separately. Clinical trials are advised to further support the current findings.

## Electronic supplementary material

Below is the link to the electronic supplementary material.

Supplementary Material 1

## Data Availability

No datasets were generated or analyzed during the current study.

## Abbreviations

UUO: Unilateral ureteral obstruction
SOF: Sorafenib
EDV: Edaravone
CKD: Chronic kidney disease
TNF-α: Tumor necrosis factor-alpha
H&E: Hematoxylin and eosin
RIPK-1: Receptor-interacting serine/threonine-protein kinase 1
RIPK-3: Receptor-interacting serine/threonine-protein kinase 3
Caspase-8: Cysteine-aspartic acid protease 8
MLKL: Mixed lineage kinase domain-like protein
Coll-1α: Collagen-1 alpha
HYP: Hydroxy proline
IL-1β: Interleukin 1beta
Nrf2: Nuclear factor erythroid 2-related factor 2
MPO: Myeloperoxidase
HO-1: Heme oxygenase 1
MDA: Malondialdehyde
GSH: Reduced glutathione
SOD: Superoxide dismutase
ECM: Extracellular matrix
ROS: Reactive oxygen species
GAPDH: Glyceraldehyde-3-phosphate dehydrogenase
RSI: Renal somatic index
