# Supplementary figures and images for: Sorafenib and edaravone protect against renal fibrosis induced by unilateral ureteral obstruction via inhibition of oxidative stress, inflammation, and RIPK-3/MLKL pathway
Source: Naunyn Schmiedebergs Arch Pharmacol. 2024 Jun 14;397(11):8961–77. doi: 10.1007/s00210-024-03146-z (PMC11522075; doi:10.1007/s00210-024-03146-z)

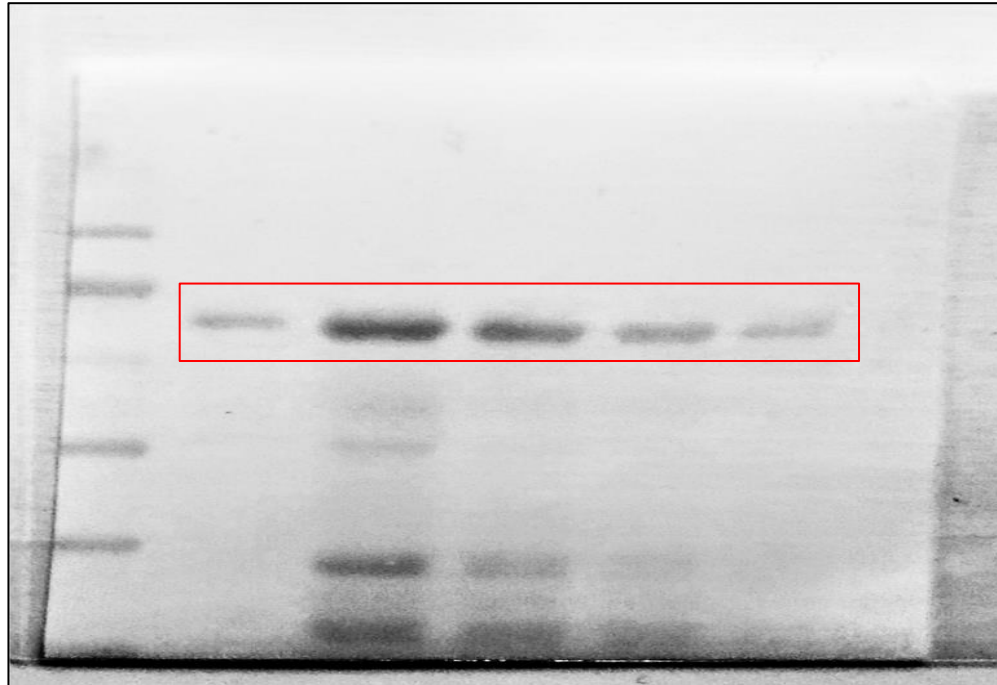

Caspase-8

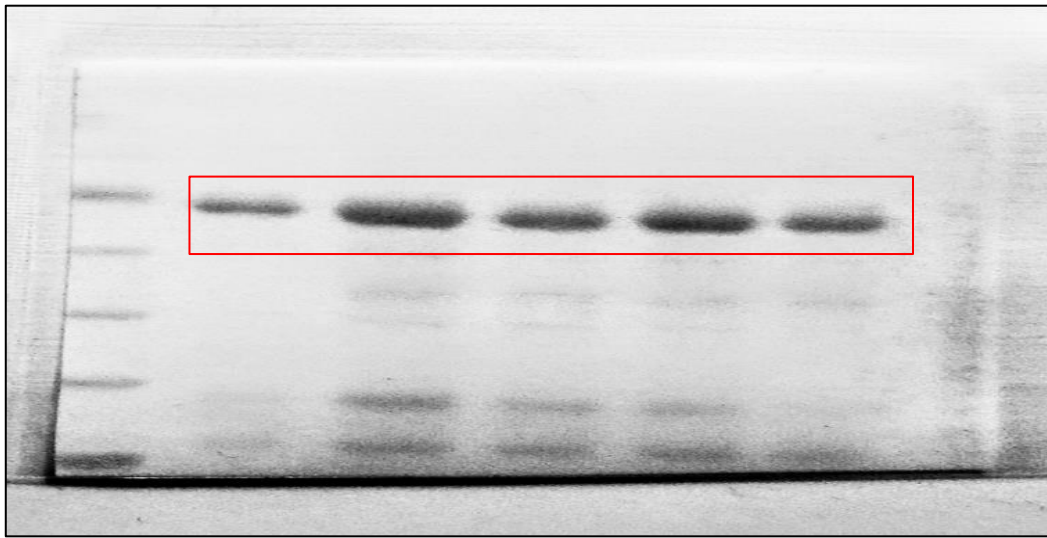

RIPK3

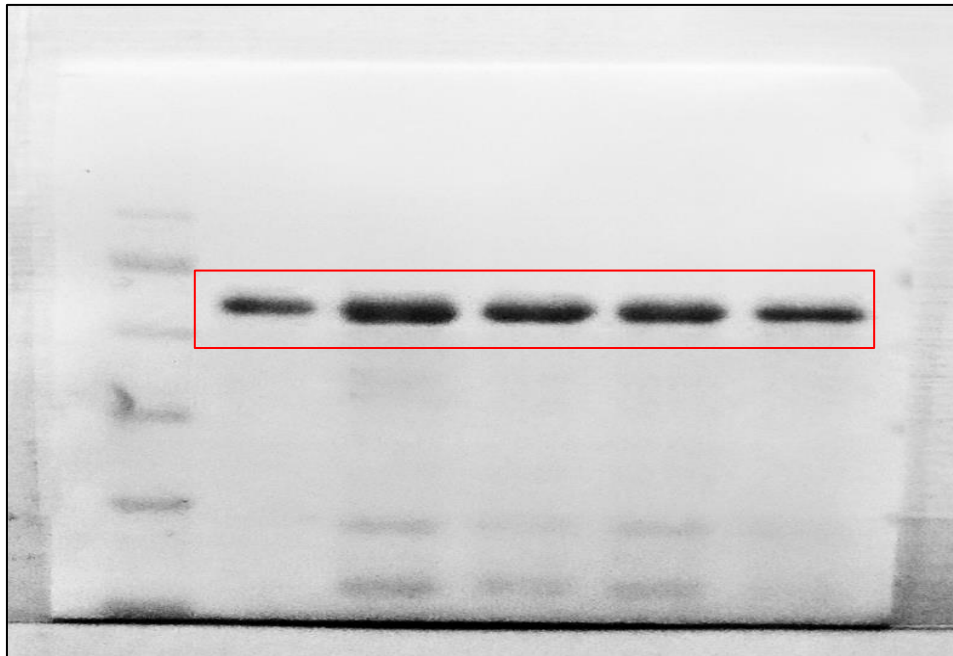

MLKL

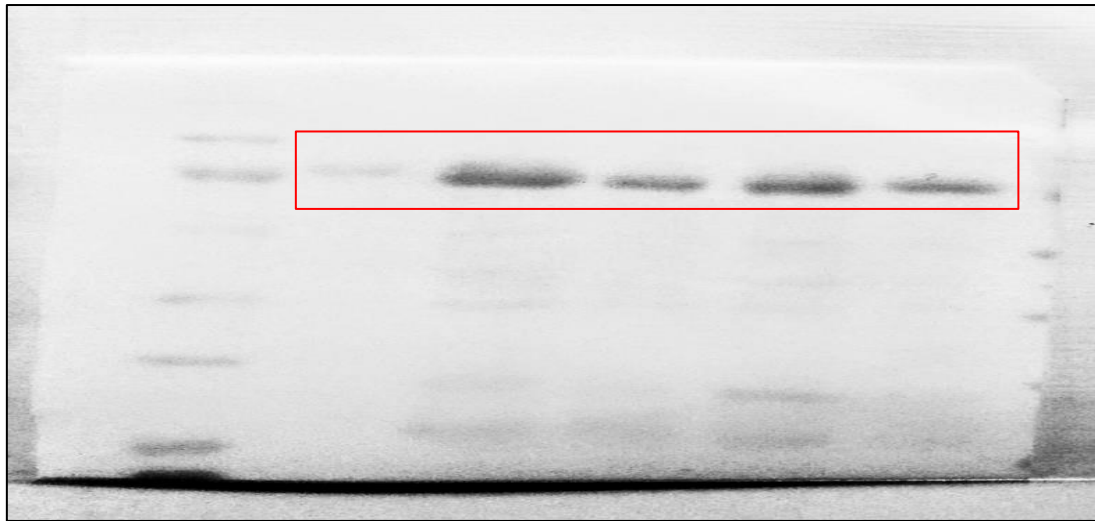

RIPK1

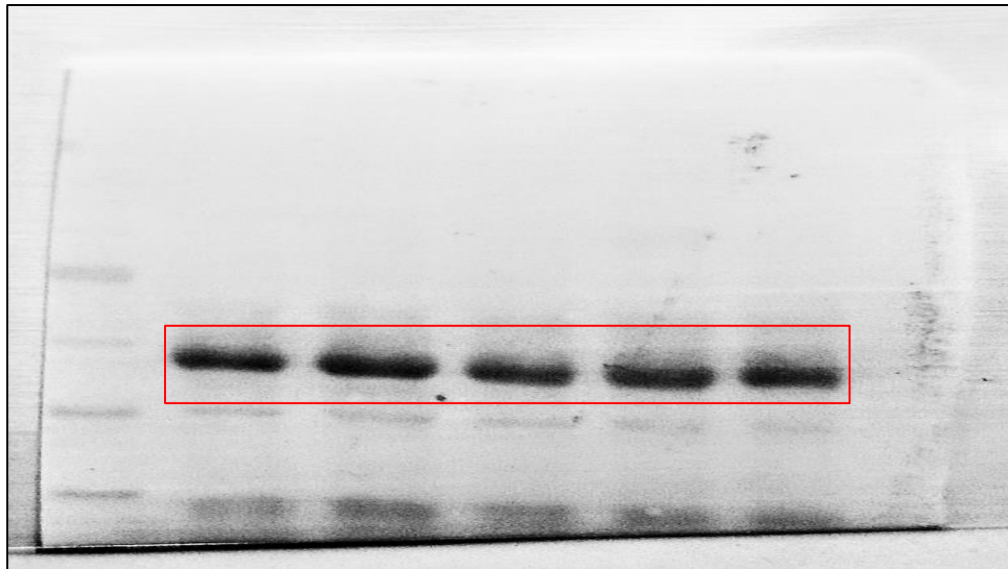

$\beta$ -actin

Supplement: Supplementary file 1 — Supplementary Material 1 [file 210_2024_3146_MOESM1_ESM.pdf]
